# Multiple Applications of Alamar Blue as an Indicator of Metabolic Function and Cellular Health in Cell Viability Bioassays

**DOI:** 10.3390/s120912347

**Published:** 2012-09-10

**Authors:** Sephra N. Rampersad

**Affiliations:** Department of Life Sciences, The University of the West Indies, West Indies, St. Augustine, Trinidad and Tobago; E-Mail: Sephra.Rampersad@sta.uwi.edu; Tel.: +1-868-662-2002 (ext. 83109); Fax: +1-868-663-5241

**Keywords:** Alamar Blue, cell viability, cytotoxicity, resazurin

## Abstract

Accurate prediction of the adverse effects of test compounds on living systems, detection of toxic thresholds, and expansion of experimental data sets to include multiple toxicity end-point analysis are required for any robust screening regime. Alamar Blue is an important redox indicator that is used to evaluate metabolic function and cellular health. The Alamar Blue bioassay has been utilized over the past 50 years to assess cell viability and cytotoxicity in a range of biological and environmental systems and in a number of cell types including bacteria, yeast, fungi, protozoa and cultured mammalian and piscine cells. It offers several advantages over other metabolic indicators and other cytotoxicity assays. However, as with any bioassay, suitability must be determined for each application and cell model. This review seeks to highlight many of the important considerations involved in assay use and design in addition to the potential pitfalls.

## 1. Introduction

Alamar Blue has been widely used over the past 50 years in studies on cell viability and cytotoxicity in a range of biological and environmental systems. It is one of the most highly referenced substances used for cytotoxicity and viability assays; PubMed records list over 200 publications citing Alamar Blue (resazurin) and cancer research and over 1,000 publications in drug screening, development and discovery. Generally, its use has been applied to various aspects of monitoring cellular health [1,2], apoptosis, cell cycle function and control [3–8], test compound toxicology in medicine and in environmental risk assessments [9–13], cytotoxicity [14–16] and antimicrobial susceptibility testing [17,18]. It has also been used to assess various bacteria, yeast and fungal species and different cell types including, but not limited to fibroblasts [19], immortalized and cancer cell lines [9,20,21], mouse and human lymphocytes [1,22], primary neuronal cell culture [4], and various animal cell lines [23,24]. As with any viability assay, optimization and assessment of compatibility must be carried out for every cell model. The objective of this review is to highlight the more important considerations involved in assay use, design and the potential pitfalls. This review gives an introduction and discussion of cell assays including their formats, chemistries, advantages and limitations, followed by a discussion of the Alamar Blue assay which covers basic chemistry, optimization parameters for the user, specific studies in relation to Alamar Blue assay optimization and comparison with other bioassays, concluding statements and a checklist of important points to consider with respect to suitability of the assay to a any given study.

## 2. Cell Viability and Cell Proliferation Assays

### 2.1. Cell Viability Assays

Morphological methods (including ultrastructural studies) require highly skilled personnel, expensive equipment and results are not usually quantifiable; cells must also be destroyed so continuous monitoring or kinetic studies are ruled out. Various cell viability and cell proliferation assays are used to determine the effect of a test compound on cells propagated *in vitro*. Indirect techniques that assess cell viability by monitoring cell membrane integrity after drug exposure, *i.e.*, dye exclusion and preferential dye uptake, also destroy or interfere with the cell's functioning and hence are terminal assays. Other assays that measure cell viability, indirectly, by quantitation of reduction of the intracellular environment using indicator metabolic markers are useful and offer fewer limitations than other methods. Assays that quantify intracellular ATP (the ATP status of cells reflects the cells' energy capacity and viability) have also been developed where the concentration of ATP in indicative of the number of viable cells in that culture. Other biomarkers that can be quantified include NADH, capases, LDH, in addition to live- and dead-cell proteases (www.promega.com). One potential drawback of some of these metabolic assays is there is no differentiation between cells that are actively dividing and those that are quiescent which may result in an over-estimation of cell number. Also, an increase in cell growth may indicate cell viability but, a decrease in viability can be interpreted as the result of either cytotoxic effects of a particular test compound or induction of sub-optimal assay conditions.

### 2.2. Cell Proliferation Assays Using Nucleotide Analogs

Cell proliferation assays monitor actively dividing cells, expressed either as the actual number of active cells or the ratio of proliferating to non-proliferating cells in culture [25–27]. Quiescent cells that are healthy but not proliferating are not detected by cell proliferation assays. Measurement of DNA synthesis has been used as a specific marker in actively dividing cells. In these assays, labeled nucleotide analogs (e.g., [^3^H]-thymidine or 5-bromo-2′-deoxyuridine [BrdU]) are added to cells and are incorporated into the replicated DNA during the S phase of the cell cycle. The amount of labeled nucleotide is then quantified by: (i) measuring the total amount of labeled DNA in the cell population, or by (ii) counting the number of labeled nuclei under a microscope. The growth rate of the cells will determine the rate of cell turnover which dictates the incubation period with the labeled nucleotide analogs. Quantitation of DNA content of cells based on radioisotopic assays are terminal, labor-intensive and present handling, storage, and disposal issues. They are also time-consuming and subject to operator errors especially for medium to high-throughput applications [25]. The clonogenic assay measures the effect of a test compound on the proliferating fraction of the population (for a review of these assays see references [25–27]).

### 2.3. A Comparison of Tetrazolium Salts as Metabolic Indicators

Tetrazolium salt reduction is a commonly used, cost-effective indirect measure of viable cell number. The most frequently used tetrazolium salts are 3-(4,5-dimethyethiazol-2-yl)-2, 5-diphenyltetrazolium bromide (MTT), sodium 3′- [1-phenylamino)-carbonyl]-3,4-tetrazolium]-bis (4-methoxy-6-nitrobenzene) sulfonic acid hydrate (XTT), and 4-[3-(4-iodophenyl)-2-(4-nitrophenyl)-2H-5-tetrazolio]-1,3-benzene disulfonate, water-soluble tetrazolium salt (WST-1). Despite their wide use and applicability, users of these assays may encounter a number of incompatibility problems depending on the study objectives. For example, compared to XTT and MTS, WST-1 is more stable, has a broader linear range and enables more rapid color development. MTT can be reduced by NADPH, FADH, FMNH, and NADH, but not by the cytochromes. Tetrazolium salts are generally cytotoxic because the formazan crystals that are produced from reduction of the salts must be solubilized with DMSO or HCl/isopropanol which destroys the cells under investigation so time-course experiments cannot be carried out [14]. MTT is not soluble in culture medium and is best suited for use with adherent cell lines; however, XTT is soluble in culture medium and is suitable for use with both non-adherent as well as adherent cell lines but XTT is not efficiently reduced without PMS (phenozine methosulfate) (www.assay.nih.gov/assay/). Thus, selection of the most appropriate tetrazolium salt as an indicator of metabolic activity is important. Alamar Blue circumvents many of the incompatibility issues described and offers many advantages over tetrazolium salts.

### 2.4. Characteristics of a Robust Viability Assay

There is a range of commercially available assays which require careful optimization according to the manufacturer's recommendations (for example, www.ab-direct.com; www.promega.com; www.invitrogen.com; www.trekds.com). It is important that the user defines what the assay has to measure, the cell model under study, and whether end-point determinations are necessary. The correlation between measurement and cell viability (taken as the number of live cells) must be determined and the potential limitations of assay chemistries are also important for application to either manual or automated high-throughput platforms. Depending on the assay chemistry, some assays may be more suited to certain cell lines or only primary cell lines or may be more applicable to adherent cells rather than cell suspensions. Further, metabolic activity can vary depending on the life-cycle of a given cell and the cell type [25,28]. Assay responsiveness is influenced by a number of factors including the culture medium *i.e.*, constituents, buffering capacity, pH, cell density, evaporation and edge-effects of the microtiter plate, incubation temperature, chemical interactions between the media components, test compound and assay chemistry, dosage and exposure time to the test compound, and linearity of the assay, test compound and assay reagent stability for time-course monitoring, instrumentation availability (spectrophotometer, fluorometer, luminometer), scalability to medium and high-throughput workflow, reproducibility of the data and level of intra- and inter-assay agreement, total cost of the assay, toxicity to the user and the environment, and whether the *in vitro* data can be used to reliably predict biological implications. It should be noted that *in vitro* data represents an estimated signal of the entire population of cells and may not always reflect an *in vivo* response. The Alamar Blue assay satisfies most of these criteria once the user carefully optimizes the reaction conditions for each cell model.

## 3. The Alamar Blue Assay

### 3.1. Assay Chemistry and Redox Principle

Alamar Blue monitors the reducing environment of the living cell. The active ingredient is resazurin (IUPAC name: 7-hydroxy-10-oxidophenoxazin-10-ium-3-one), also known as diazo-resorcinol, azoresorcin, resazoin, resazurine, which is water-soluble, stable in culture medium, is non-toxic and permeable through cell membranes. Continuous monitoring of cells in culture is therefore permitted. Resazurin was first used to assess bacterial or yeast contamination in milk by Pesch and Simmert in 1929. It is a blue non-fluorescent dye that is reduced to the pink-colored, highly fluorescent resorufin. Resazurin solution is highly dichromatic based on Kreft's dichromaticity index (DI) [29]. The dye acts as an intermediate electron acceptor in the electron transport chain without interference of the normal transfer of electrons [9]. The oxidation-reduction potential of Alamar Blue is +380 mV at pH 7.0, 25 °C. Alamar Blue, therefore, can be reduced by NADPH (E_o_ = 320 mV), FADH (E_o_ = 220 mV), FMNH (E_o_ = 210 mV), NADH (E_o_ = 320 mV), as well as the cytochromes (E_o_ = 290 mV to +80 mV). As the indicator dye accepts electrons, it changes from the oxidized, non-fluorescent, blue state to the reduced, fluorescent, pink state [9]. In addition to mitochondrial reductases, other enzymes (such as the diaphorases (EC 1.8.1.4, dihydrolipoamine dehydrogenase [30]), NAD(P)H:quinone oxidoreductase (EC 1.6.99.2) [31] and flavin reductase (EC 1.6.99.1) [32] located in the cytoplasm and the mitochondria may be able to reduce Alamar Blue. Hence, Alamar Blue reduction may signify an impairment of cellular metabolism and is not necessarily specific to interruption of electron transport and mitochondrial dysfunction [33]. This change from oxidized to reduced state allows flexibility of detection where measurements can be quantitative as colorimetric and/or fluorometric readings (the latter being more sensitive) or qualitative as a visible change in color indicating presence or absence of viable cells. Spectrophotometric absorbance is taken at two wavelengths (570 and 600 nm or 540 and 630 nm, although other filters can be used with a correction factor to be incorporated in calculations). Absorbance values will vary according to microtiter plate (flat-bottomed or rounded and manufacturer). Fluorescence signals are measured at an excitation wavelength at 530–560 nm and an emission wavelength at 590 nm.

### 3.2. Optimization Parameters and Assay Responsiveness

The optimum pH of the assay is ranges between 7.0 and 7.4, therefore, culture medium must have buffering capacity. The optimum incubation temperature is 37 °C and plates must therefore, be sealed to prevent evaporation. Assays must be carried out at a uniform temperature to ensure reproducibility of all wells and across a single plate. Alamar Blue is photosensitive and the incubations must be done in the dark. The culture medium and the test compound themselves should not interact with the assay chemistry. Negative and positive controls must be empirically determined to ensure that there are no non-specific interactions with assay chemistry which would result in artifacts or false positive signals. The incubation time and cell density must be empirically determined and standardized as low cell density means slower growth and lower than expected levels of dye reduction. The end-point of the assay depends on the cell density used. Generally, it is recommended that the cells should be in the exponential stage of growth. It is also preferred that the culture medium should be synthetic and defined, however, it must also allow sufficient growth so that stimulatory or inhibitory effects of test compounds are not exaggerated or underestimated.

Bleaching out of the pink color of the reduced state of the dye (quenching of fluorescence) usually occurs after very prolonged incubation times due to the formation of a colorless product called dihydroresorufin. Exhaustion of the buffering capacity of the dye's formulation may explain the formation of this product. 10% fetal bovine serum (FBS) and bovine serum albumin (BSA) may also cause quenching of the assay [9,34]. Phenol red shifts all values 0.03 units higher than without phenol red, but it does not otherwise affect assay chemistry. Microbial contamination will reduce the dye and lead to false positive signals hence, the assay must be carried out under aseptic conditions and the medium may require antibiotics to eliminate microbial contamination.

## 4. Specific Studies in Relation to Suitability of the Alamar Blue Assay and Comparison with other Bioassays

The various parameters that influence assay responsiveness are particularly important in studies of anti-microbial sensitivity in different micro-organisms. Alamar Blue has been used extensively in biomedical research to assess the relative susceptibilities of a number of pathogens to anti-microbial compounds [15,35–42], including multi-drug screening of various clinically important pathogenic yeasts and filamentous fungi (including *Aspergillus* spp., *Candida* spp.) with high levels of agreement with the recommended reference methods of the NCCLS [18,37,41,42].

Similarly, there have been numerous publications that describe the successful use of Alamar Blue in drug screening for bacterial pathogens including but not limited to *Mycobacterium* spp., *Staphylococcus* spp., *Enterococcus* spp. and *Pseudomonas* spp [17,43–46]. For *Mycobacterium* spp., Alamar Blue test results had a high level of agreement with the BACTEC systems [17,38–39]. However, one study compared seven bioassays with different assay chemistries nitrate reductase assay (NRA), microscopic observation drug susceptibility (MODS), Mycobacterium Growth Indicator Tube 960 (MGITTM 960), Genotype^®^ MTBDR*plus*, Alamar Blue, MTT and resazurin assays for the detection of multi-drug susceptibilities of 31 well-characterized Ugandan strains of *M. tuberculosis* [47]. It was reported that Alamar Blue, MTT and resazurin assays had variable sensitivity and specificity compared with the other assays.

Recently, the Alamar Blue assay has been used to study fungicide sensitivity of five plant pathogens, *Monilinia fructicola* [48], *Botrytis cinerea* [49], *Verticillium dahliae* [50], *Colletotrichum* spp. [51] and *Alternaria alternata* [52]. Conventional methods of testing susceptibility of fungi to different classes of fungicides include mycelial growth assays of single-spore isolates on amended agar (AA). These methods are laborious, time consuming, require considerable amount of media and lab space, are not amenable to automation for high-throughput screening, and may not identify intermediate sensitivity to a particular fungicide [53]. Further, there may be problems of diffusion of the test compound and the agar, there may be limited contact between the fungus and the amended agar surface, and responses for broth cultures may be different from those on semi-solid medium due to differences in oxygen tension. The microdilution method circumvents some of the problems encountered by the AA test but results may be inaccurate because spectrophotometric measurements include both viable and nonviable (dead) spores. Various researchers have also developed antifungal sensitivity assays based on flow cytometry using a number of different dyes, which can be toxic to the user and to the cells hence, time-course experiments cannot be conducted [54]. Methods that require the use of a hemacytometer to carry out spore counts are laborious and prone to variable results when working with numerous isolates and replicates. The use of an optimized Alamar Blue assay as a quantitative colorimetric assay in determining the relative efficacies of differently acting fungicides on spore viability has been described for different plant pathogenic fungi [49,51]. The assay can also be utilized as a qualitative one where presence or absence of a color change of the dye indicates presence or absence of viable spores [52]. The Alamar Blue assay in conjunction with amended agar assays would be able to discriminate among test compounds that affect spore viability, those that only suppress mycelial growth or both [51].

Methods for high-throughput screening of anti-biofilm compounds are needed. In such assays, Alamar Blue has been shown to be a more reproducible and cheaper redox indicator than XTT [55] and is useful in identifying resistance-compromised mutants and identification of compounds with anti-biofilm activity. The assay has been used to identify antimicrobials with enhanced efficacy against certain clinically important bacterial and fungal biofilms [56–60].

The Alamar Blue assay has been exploited for monitoring immune cell proliferation and function. Immune cells including lymphocytes, monocytic macrophage cell lines, interleukin-dependent cytotoxic T cell lines, dendritic cells and myeloma cells have been monitored by Alamar Blue assays [61–63], since the assay does not require cell lysis and continuous monitoring through time-course experiments is possible [64,65]. It was also used in time-course experiments to investigate immuno-modulatory effects [66,67]. The specific advantages of the Alamar Blue assay over the [^3^H]thymidine assay for lymphocyte proliferation studies are: (i) non-radioactive, (ii) ease of use, (iii) lower cost, (iv) not labor intensive, (v) rapid assessment of proliferation of large number of samples; (vi) non-toxic; (vii) useful in determining the kinetics of cell growth of hybridomas, and (viii) no interference between secreted of antibodies of a given hybridoma cell line and assay chemistry.

## 5. Alamar Blue Compatibility and Assay Chemistry Interactions

As with any bioassay, suitability must be determined for each application and cell model. While Alamar Blue has been used extensively in cell viability and cytotoxicity studies, its use in monitoring cell proliferation may be limited to studying specific cell models.

### 5.1. Selecting a Cell Model

The effects of indomethacin on tendon-derived cell proliferation using the Alamar Blue assay was studied [28]. It was demonstrated that tendons appear to contain two subpopulations of cells; one subpopulation with apparently normal metabolic activity and a second subpopulation of cells with low levels of mitochondrial enzymes and subsequently low levels of oxidative metabolism. Although it was also concluded that the Alamar Blue dye had no toxic effect on these cells, because of the differential metabolic rate of tendon-derived cells, reduction of Alamar Blue dye may appear to be non-linear [28]. These findings suggest that the Alamar Blue assay may not be appropriate for studying proliferation in tendon-derived cells.

Cell proliferation was assessed by comparing the results of an Alamar Blue assay and two DNA quantification assays, CyQuant and PicoGreen, on two human cancer cell lines and two human primary cell sources [25]. It was found that the metabolic assays may not accurately reflect cellular proliferation rates due to a non-linear correlation between dye reduction and cell number that led to an over-estimation of cell number. Although the Alamar Blue assay is a simple, fast and sensitive assay it may not be as useful in assessing cell proliferation in these cell lines and, therefore, may be more valuable to cytotoxicity testing.

Investigation of anti-proliferative compounds or cytotoxic therapeutic agents to treat malignant cells or aberrant cell proliferation is an important aspect of drug development. Viability assays that can quantify biological activity and differentiate the relative stability and toxicity of various test compounds are the most valuable. As indicated earlier, one of the more critical assumptions of such quantitative viability assays is that cell number and drug concentration share a linear and inversely proportional relationship. This assumption is violated in cases of highly variable cell number in time-course experiments because of a number of factors, for example: (i) where the cell line itself has irregular growth properties, (ii) where a smaller number of responsive non-proliferating cells is masked by a larger number of non-responsive proliferating cells, (iii) where the test compound affects cellular aggregation or adhesion-both of which can indirectly affect cell proliferation, and (iv) where the test compound causes apoptosis or changes the cell cycle which would result in a nonlinear relationship between drug dose and cell number. In some cases where feasible, it may be necessary to normalize the data to cell number by using second assay (e.g., TruCount beads or a hemocytometer) [68].

Resazurin caused oxidative stress that lead to autophagy and cell death through cellular production of reactive oxygen species (ROS) and mitochondrial impairment in HL-60 and ‘Jurkat’ leukemia cells [69]. The dye itself caused a reduction in leukemia cell proliferation which has important implications in toxicity and chemo-resistance screening. This emphasizes the importance of empirically testing positive and negative controls for the assay to ensure no non-specific interaction effects.

The development of an Alamar Blue assay to evaluate plant cell viability and/or proliferation in tomato cell suspension was studied [70]. Comparisons were made with the more commonly used 2,3,5-triphenyltetrazolium chloride (TTC) assay. Compared to the TTC viability assay, the Alamar Blue assay was: (i) more rapid since no organic extraction of the product was required, (ii) non-toxic to the cells which facilitated downstream analysis of the same cell culture, (iii) different quantification methods can be used, and (iv) more sensitive at low percentages of cell viability. However, the disadvantages of the AB assay included a lower substrate capacity than the TTC assay.

### 5.2. Interaction Effects

Some of the reductases involved in reduction of Alamar Blue are present in subcellular components and not only in the mitochondria [33,71] which implies that Alamar Blue reduction may not be entirely due to changes in mitochondrial function. Depending on the objectives of any given study, it may be necessary to determine the importance of mitochondria-restricted Alamar Blue reduction for each cell model. Knowing the contribution of mitochondrial reductases to Alamar Blue reduction may be important to establishing the predictive ability of *in vitro* data [72].

Interaction effects and assay compatibility problems have been specifically demonstrated in screening bioengineered nanomaterials. Engineered nanomaterials vary in diversity and complexity of the types of materials and have different physicochemical properties [73]. As a result, it has been recommended that a diverse portfolio of cell viability and cytotoxicity assays (including the Alamar Blue assay) with different chemistries and detection mechanisms should be used for the assessment of nanoparticles or nanomaterials [74,75]. One reason is that some nanoparticles may interact with assay chemistry [76] and there is difficulty in determining *in vitro* effects [77]. Further, the composition of the culture medium and the nanomaterials itself, use of dispersants or other additives, the proliferation rate and the type of the cells used in the assay may also have an effect on assay outcome [76,78].

## 6. Conclusions

Accurate prediction of the adverse effects of test compounds on living systems, detection of toxic thresholds, and expansion of experimental data sets to include multiple toxicity end-point analysis are required for any robust screening regime. The Alamar Blue assay provides accurate time-course measurements, has high sensitivity and linearity, involves no cell lysis, is ideal for use with post-measurement functional assays, is flexible as it can be used with different cell models, is scalable and can be used with fluorescence- and/or absorbance-based instrumentation platforms, and finally, it is non-toxic, non-radioactive and is safe for the user, and the environment. *In vitro* bioassays may not be highly predictive of *in vivo* effects and there may be a need for the development of multiple parameters which would assist in translating *in vitro* data into meaningful *in vivo* effects [72]. There is a profusion of data that supports the use of Alamar Blue as a cell viability assay, however, numerous studies have adopted a combinatorial approach to cell viability and cytotoxicity screening and, therefore, utilized different assay chemistries in order to produce the most meaningful and representative *in vitro* result.

Important Points to Consider with Respect to Suitability of the Alamar Blue Assay for a Specific Application

- Study objective: specific impairment of cytosolic *vs.* mitochondrial reductases important?
- Cell model: cell type, adherent/non-adherent cells, cell density, cell line and age of cells under study
- Other studies demonstrated success with the cell model under investigation?
- Availability and type of instrumentation: fluorometric or colorimetric or both?
- Data recovery: indirect qualitative and/or quantitative?
- End-point determinations important?
- Assay responsiveness must be assessed by evaluation the following: culture medium *i.e.*, constituents, buffering capacity, pH, evaporation and edge-effects, microtiter plate: flat or round-bottomed, manufacturer, incubation temperature and time, chemical interactions between the media components, test compound and assay chemistry compatibility, dosage and exposure time to the test compound, linearity of the assay, negative and positive controls
- Need for a second assay to directly measure cell number?
- Time-course monitoring required?
- Storage and stability of the reagent
- Reproducibility of the data and level of intra- and inter-assay agreement
- Total cost of the assay
- Can biological implications be reliably predicted from *in vitro* data?

## References

[1] Ahmed S.A., Gogal R.M., Walsh J.E. (1994). A new rapid and simple non-radioactive assay to monitor and determine the proliferation of lymphocytes: An alternative to [3H] thymidine incorporation assay. J. Immunol. Meth..

[2] O'Brien J., Wilson I., Orton T., Pognan F. (2000). Investigation of Alamar Blue (resazurin) fluorescent dye for the assessment of mammalian cell cytotoxicity. Eur. J. Biochem..

[3] Farinelli S.E., Greene L.A. (1996). Cell cycle blockers mimosine, ciclopirox, and deferoxamine prevent the death of PC12 cells and postmitotic sympathetic neurons after removal of trophic support. J. Neurosci..

[4] White M.J., Di Caprio M.J., Greenberg D.A. (1996). Assessment of neuronal viability with Alamar Blue in cortical and granule cell cultures. J. Neurosci. Meth..

[5] Mizuno R., Oya M., Shiomi T., Marumo K., Murai M. (2004). Inhibition of MKP-1 expression potentiates JNK related apoptosis in renal cancer cells. J. Urol..

[6] Durrant D., Richards J.E., Walker W.T., Baker K.A., Simoni D., Lee R.M. (2008). Mechanism of cell death induced by cis-3,4′,5-trimethoxy-3′-aminostilbene in ovarian cancer. Gynecol. Oncol..

[7] Yang S.Y., Sales K.M., Fuller B.J., Seifalian A.M., Winslet M.C. (2008). Inducing apoptosis of human colon cancer cells by an IGF-I D domain analogue peptide. Mol. Cancer.

[8] Yao K., Youn H., Gao X., Huang B., Zhou F., Li B., Han H. (2012). Casein kinase 2 inhibition attenuates androgen receptor function and cell proliferation in prostate cancer cells. Prostate.

[9] Page A.B., Page A.M., Noel C. (1993). A new fluorimetric assay for cytotoxicity measurements *in vitro*. Int. J. Oncol..

[10] Takahasi T., Maruyama W., Deng Y., Dostert P., Nakahara D., Niwa T., Ohta S., Naoi M. (1997). Cytotoxicity of endogenous isoquinolines to human dopaminergic neuroblastoma SH-SY5Y cells. J. Neural. Transm..

[11] Schirmer K., Dixon D.G., Greenberg B.M., Bols N.C. (1998). Ability of 16 priority PAHs to be directly cytotoxic to a cell line from the rainbow trout gill. Toxicology.

[12] Desaulniers D., Leingartner K., Zacharewski T., Foster W.G. (1998). Optimization of a MCF-E3 cell proliferation assay and effects of environmental pollutants and industrial chemicals. Toxicol. In Vitro.

[13] Hamid R., Rotshteyn Y., Rabadi L., Parikh R., Bullock P. (2004). Comparison of Alamar Blue and MTT assays for high through-put screening. Toxicol. In Vitro.

[14] Mosmann T. (1983). Rapid colorimetric assay for cellular growth and survival: Application to proliferation and cytotoxicity assays. J. Immunol. Meth..

[15] Nociari M.M., Shalev A., Benias P., Russo C. (1998). A novel one-step, highly sensitive fluorometric assay to evaluate cell-mediated cytotoxicity. J. Immunol. Meth..

[16] O'Brien J., Wilson I., Orton T., Pognan F. (2000). Investigation of the Alamar Blue (resazurin) fluorescent dye for the assessment of mammalian cell cytotoxicity. Eur. J. Biochem..

[17] Yajko D.M., Madej J.J., Lancaster M.V., Sanders C.A., Cawthon V.L., Gee B., Babst A., Hadley W.K. (1995). Colorimetric method for determining MICs of antimicrobial agents for Mycobacterium tuberculosis. J. Clin. Microbiol..

[18] Lozano-Chiu M., Lancaster M.V., Rex J.H. (1998). Evaluation of a colorimetric method for detecting amphotericin b-resistant candida isolates. Diagn. Microbiol. Infec. Dis..

[19] Voytik-Harbin S.L., Brightman A.O., Waisner B., Lamar C.H., Badylak S.F. (1998). Application and evaluation of the Alamar Blue assay for cell growth and survival of fibroblasts. In Vitro Cell Dev. Biol. Anim..

[20] Nakayama G.R., Caton M.C., Nova M.P., Parandoosh Z. (1997). Assessment of the Alamar Blue assay for cellular growth and viability *in vitro*. J. Immunol. Meth..

[21] Al-Nasiry S., Geusens N., Hanssens M., Luyten C., Pijnenborg R. (2006). The use of Alamar Blue assay for quantitative analysis of viability, migration and invasion of choriocarcinoma cells. Hum. Rep..

[22] De Fries R., Mistuhashi M. (1995). Quantification of mitogen induced human lymphocyte proliferation: Comparison of Alamar Blue assay to 3H-thymidine incorporation assay. J. Clin. Lab. Anal..

[23] Dayeh V.R., Bols N.C., Schirmer K., Lee L.E.J. (2003). The use of fish-derived cell lines for investigation of environmental contaminants. Curr. Protoc. Toxicol..

[24] Davoren M., Ní Shúilleabháin S., Hartl M.G., Sheehan D., O'Brien N.M., O'Halloran J., van Pelt F.N., Mothersill C. (2005). Assessing the potential of fish cell lines as tools for the cytotoxicity testing of estuarine sediment aqueous elutriates. Toxicol. In Vitro.

[25] Quent V.M.C., Loessner D., Friis T., Reichert J.C., Hutmacher D.W. (2010). Discrepancies between metabolic activity and DNA content as tool to assess cell proliferation in cancer research. J. Cell Mol. Med..

[26] Stoddart M.J. (2011). Cell viability assays: Introduction, mammalian cell viability. Meth. Mol. Biol..

[27] Longo-Sorbello G.S.A., Saydam G., Banerjee D., Bertino J.R., Julio C., Nigel C., Kai S., Small J., Hunter T., David S. (2005). Chapter 38: Cytotoxicity and cell growth assays. Cell Biology a Laboratory Handbook.

[28] Mallick E., Scutt N., Scutt A., Rolf C. (2009). Passage and concentration-dependent effects of Indomethacin on tendon derived cells. J. Orthop. Surg. Res..

[29] Kreft S., Kreft M. (2009). Quantification of dichromatism: A characteristic of color in transparent materials. J. Opt. Soc. Am. A..

[30] Matsumoto K., Yamada Y., Takahashi M., Todoroki T., Mizoguchi K., Misaki H., Yuki H. (1990). Fluorometric determination of carnitine in serum with immobilized carnitine dehydrogenase and diaphorase. J. Clin. Chem..

[31] Belinsky M., Jaiswal A.K. (1993). NAD(P)H: quinone oxidoreductase1DT-diaphorase expression in normal and tumor tissues. Cancer Metastasis Rev..

[32] Chikuba K., Yubisui T., Shirabe K., Takeshita M. (1994). Cloning and nucleotide sequence of a cDNA of the human erythrocyte NADPH-flavin reductase. Biochem. Biophys. Res. Commun..

[33] Moon T.W., Mommsen T.P. (2005). Biochemistry and molecular biology of fishes. Environ. Toxicol..

[34] Goegan P., Johnson G., Vincent R. (1995). Effects of serum protein and colloid on the Alamar Blue assay in cell cultures. Toxicol. In Vitro.

[35] Jorgensen J.H. (1993). Selection criteria for an antimicrobial susceptibility testing system. J. Clin. Microbiol..

[36] Pfaller M.A., Barry A.L. (1994). Evaluation of a novel colorimetric broth microdilution method for antifungal susceptibility testing of yeast isolates. J. Clin. Microbiol..

[37] Collins L., Franzblau S.G. (1997). Microplate Alamar Blue assay *versus* BACTEC 460 system for high-throughput screening of compounds against *Mycobacterium tuberculosis* and *Mycobacterium avium*. Antimicrob. Agents Ch..

[38] Franzblau S.G., Witzig R.S., McLaughlin J.C., Torres P., Madico G., Hernandez A., Degnan M.T., Cook M.B., Quenzer V.K., Ferguson R.M. (1998). Rapid, low-technology MIC determination with clinical *Mycobacterium tuberculosis* isolates by using the microplate Alamar Blue assay. J. Clin. Microbiol..

[39] Cuenca-Estrella M., Rodriguez-Tuleda J.L. (2001). Present status of the detection of antifungal resistance: The perspective from both sides of the ocean. J. Clin. Micobiol. Infect..

[40] Meletiadis J., Mouton J.W., Meis J.F.G.M., Bouman B.A., Verweij P.E. (2002). Comparison of the Etest and the sensititre colorimetric methods with the NCCLS proposed standard for antifungal susceptibility testing of *Aspergillus* species. J. Clin. Microbiol..

[41] Reis R.S., Ivan N., Lourenço S.L.S., Fonseca L.S., Lourenço M.C.S. (2004). Comparison of flow cytometric and Alamar Blue tests with the proportional method for testing susceptibility of *Mycobacterium tuberculosis* to rifampin and isoniazid. J. Clin. Microbiol..

[42] McBride J., Ingram P.R., Henriquez F.L., Roberts C.W. (2005). Development of colorimetric microtiter plate assay for assessment of antimicrobials against *Acanthamoeba*. J. Clin. Microbiol..

[43] Yamaguchi H., Uchida K., Nagino K., Matsunaga T. (2002). Usefulness of a colorimetric method for testing antifungal drug susceptibilities of Aspergillus species to voriconazole. J. Infect. Chemother.

[44] Zabransky R.J., Dinuzzo A.R., Woods G.L. (1995). Detection of vancomycin resistance in enterococci by the alamar mic system. J. Clin. Microbiol..

[45] Hoffman L.R., Déziel E., D'Argenio D.A., Lépine F., Emerson J., McNamara S., Gibson R.L., Ramsey B.W., Miller S.I. (2006). Selection for *Staphylococcus aureus* small-colony variants due to growth in the presence of *Pseudomonas aeruginosa*. Proc. Natl. Acad. Sci..

[46] Martin A., Portaels F., Palomino J.C. (2007). Colorimetric redox-indicator methods for the rapid detection of multidrug resistance in *Mycobacterium tuberculosis*: A systematic review and meta-analysis. J. Antimicrob. Chemother.

[47] Bwanga F., Joloba M.L., Haile M., Hoffner S. (2010). Evaluation of seven tests for the rapid detection of multidrug-resistant tuberculosis in Uganda. Int. J. Tuberc. Lung Dis..

[48] Pijls C.F.N., Shaw M.W., Parker A. (1994). A rapid test to evaluate *in vitro* sensitivity of *Septoria tritici* to flutriafol, using a microtiter plate reader. Plant Pathol..

[49] Cox K.D., Quello K., Deford R.J., Beckerman J.L. (2009). A rapid method to quantify fungicide sensitivity in the brown rot pathogen *Monilinia fructicola*. Plant Dis..

[50] Pelloux-Prayer A.L., Priem B., Joseleau J.P. (1998). Kinetic evaluation of conidial germination of *Botrytis cinerea* by a spectrofluorometric method. Mycol. Res..

[51] Rampersad S.N. (2011). A rapid colorimetric microtiter bioassay to evaluate fungicide sensitivity among *Verticillium dahliae* isolates. Plant Dis..

[52] Rampersad S.N., Teelucksingh L.D. (2012). Differential responses of *Colletotrichum gloeosporioides* and *C. truncatum* isolates from different hosts to multiple fungicides based on two assays. Plant Dis..

[53] Vega B., Liberti D., Harmon P.F., Dewdney M.M. (2012). A rapid resazurin-based microtiter assay to evaluate QoI sensitivity for *Alternaria alternata* isolates and their molecular characterization. Plant Dis..

[54] Fai P., Grant A. (2009). A rapid resazurin bioassay for assessing the toxicity of fungicides. Chemosphere.

[55] Benoit M.R., Conant C.G., Ionescu-Zanetti C., Schwartz M., Matin A. (2010). New device for high-throughput viability screening of flow biofilms and applied. Environ. Microbiol..

[56] Repp K.K., Menor S.A., Pettit R.K. (2007). Microplate Alamar Blue assay for susceptibility testing of Candida albicans biofilms. Med. Mycol..

[57] Dorocka-Bobkowska B., Düzgüneº N., Konopka K. (2009). AmBisome and Amphotericin B inhibit the initial adherence of Candida albicans to human epithelial cell lines, but do not cause yeast detachment. Med. Sci. Monit..

[58] Pettit R.K., Weber C.A., Pettit G.R. (2009). Application of a high throughput Alamar Blue biofilm susceptibility assay to Staphylococcus aureus biofilms. Ann. Clin. Microbiol. Antimicrob..

[59] Naves P., Del Prado G., Ponte C., Soriano F. (2010). Differences in the *in vitro* susceptibility of planktonic and biofilm-associated Escherichia coli strains to antimicrobial agents. J. Chemother.

[60] Davies D. (2003). Understanding biofilm resistance to antibacterial agents. Nat. Rev. Drug Discov..

[61] Song H.F., Zhou J., Pan K., Wang Q.J., Wang H., Huang L.X., Li Y.Q., Xia J.C. (2008). Antitumor effects and mechanisms of a dendritic cell vaccine which silenced SOCS1 by siRNA, stimulated by OK-432 and pulsed with lysate of HepG2 cells. Ai Zheng.

[62] Tong H.X., Lu C.W., Wang L.W., Wang Q.S., Zhang J.H. (2010). Role of caspase-8 in TRAIL-induced apoptosis of neuroblastoma cell lines. Chin. J. Contemp. Pediatr..

[63] Zhang J., Wu L., Feng M.X., Sexton P., Bai C.X., Qu J.M., Merrilees M., Black P. (2011). N Pulmonary fibroblasts from COPD patients show an impaired response of elastin synthesis to TGF-β1. Respir. Physiol. Neurobiol..

[64] Zhi-Jun Y., Sriranganathan N., Vaught T., Arastu S.K., Ahmed S.A. (1997). A dye-based lymphocyte proliferation assay that permits multiple immunological analyses: mRNA, cytogenetic, apoptosis, and immunophenotyping studies. J. Immunol. Methods.

[65] Kwack K., Lynch R.G. (2000). A new non-radioactive method for IL-2 bioassay. Mol. Cells.

[66] Bertolo A., Thiede T., Aebli N., Baur M., Ferguson S.J., Stoyanov J.V. (2011). Human mesenchymal stem cell co-culture modulates the immunological properties of human intervertebral disc tissue fragments *in vitro*. Eur. Spine J..

[67] Mahboob H.Q., Garvy B.A. (2001). Neonatal t cells in an adult lung environment are competent to resolve *Pneumocystis carinii* pneumonia. J. Immunol..

[68] McCutcheon K., Fei D. Comparing Quantitative Viability Bioassays: An Evaluation of MTT, alamarBlue™, and Guava^®^ ViaCount^®^ Methods. Application Note: Comparing Quantitative Viability Bioassays.

[69] Erikstein B.S., Hagland H.R., Nikolaisen J., Kulawiec M., Singh K.K., Gjertsen B.T., Tronstad K.J. (2010). Cellular stress induced by resazurin leads to autophagy and cell death via production of reactive oxygen species and mitochondrial impairment. J. Cell Biochem..

[70] Byth H.-A., Bongani I.M., Ian A., Bornman D.L. (2001). Assessment of a simple, non-toxic Alamar Blue cell survival assay to monitor tomato cell viability. Phytochem. Anal..

[71] Gonzalez R.J., Tarloff J.B. (2001). Evaluation of hepatic subcellular fractions for Alamar Blue and MTT reductase activity. Toxicol. In Vitro.

[72] James M., McKim J.M. (2010). Building a tiered approach to *in vitro* predictive toxicity screening: A focus on assays with *in vivo* relevance. Comb. Chem. High Throughput Screen.

[73] Lewinski N., Colvin V., Drezek R. (2008). Cytotoxicity of nanoparticles. Small.

[74] Jones C.F., Grainger D.W. (2009). *In vitro* assessments of nanomaterial toxicity. Adv. Drug Deliv. Rev..

[75] Geys J., Nemery B., Hoet P.H. (2010). Assay conditions can influence the outcome of cytotoxicity tests of nanomaterials: Better assay characterization is needed to compare studies. Toxicol. In Vitro.

[76] Monteiro-Riviere N.A., Inman A.O., Zhang L.W. (2009). Limitations and relative utility of screening assays to assess engineered nanoparticle toxicity in a human cell line. Toxicol. Appl. Pharmacol..

[77] Atala A. (2004). Tissue engineering and regenerative medicine: concepts for clinical application. Rejuvenation Res..

[78] Lai D.Y. (2012). Toward toxicity testing of nanomaterials in the 21st century: A paradigm for moving forward. WIREs Nanomed. Nanobiotechnol.

